# Molecular Networks and Key Regulators Underlying Resilience of the Human Brain to Aging and Dementia

**DOI:** 10.3390/biom16070992

**Published:** 2026-07-06

**Authors:** Lei Guo, Nicholas Grimaldi, Minghui Wang, Lap Ho, Ben Shackleton, Ryan Neff, Erming Wang, Zhidong Tu, Sam Gandy, Vahram Haroutunian, Michelle E. Ehrlich, Charles Mobbs, Bin Zhang

**Affiliations:** 1 Department of Genetics and Genomic Sciences, Icahn School of Medicine at Mount Sinai, 1425 Madison Avenue, New York, NY 10029, USA; 2 Mount Sinai Center for Transformative Disease Modeling, Icahn School of Medicine at Mount Sinai, 1425 Madison Avenue, New York, NY 10029, USA; 3 Icahn Genomics Institute, Icahn School of Medicine at Mount Sinai, 1425 Madison Avenue, New York, NY 10029, USA; 4 Nash Family Department of Neuroscience, The Friedman Brain Institute, Icahn School of Medicine at Mount Sinai, 1425 Madison Avenue, New York, NY 10029, USA; 5 Department of Neurology, The Friedman Brain Institute, Icahn School of Medicine at Mount Sinai, 1425 Madison Avenue, New York, NY 10029, USA; 6 Department of Psychiatry, Icahn School of Medicine at Mount Sinai, 1425 Madison Avenue, New York, NY 10029, USA; 7 Alzheimer’s Disease Research Center, Icahn School of Medicine at Mount Sinai, 1399 Park Ave, New York, NY 10029, USA; 8 James J Peters VA Medical Center, 130 West Kingsbridge Road, Bronx, NY 10468, USA; 9 Department of Pediatrics, Icahn School of Medicine at Mount Sinai, 1425 Madison Ave, New York, NY 10029, USA

**Keywords:** brain resilience, gene coexpression network, dementia, Alzheimer’s disease

## Abstract

Alzheimer’s disease (AD) is an aging-related neurodegenerative disease characterized by an initial memory impairment that progresses to a widespread cerebrocortical failure, culminating in death. Understanding the molecular mechanisms that protect brain function during aging may help reveal novel targets for the development of effective treatments for the memory and cognitive deficits associated with AD. In this study, we analyzed a gene expression dataset generated from the prefrontal cortices of individuals showing no neurological or cognitive abnormalities. The gene expression profiles were used to identify candidate protective genes. We then compared the expression patterns of these genes in aging with their expression patterns in AD, thereby enabling us to pinpoint the genes that potentially contribute to brain resilience that delays or prevents aging-related dementia. We selected seven genes that are potentially protective for aging and AD, and have known homologues in *Caenorhabditis elegans* (*C. elegans*). Among these genes, *SRPK2*, *AAK1*, *EFR3A* and *MAPK10* were previously implicated in attenuating AD-related cognitive decline. Our experiments demonstrated that all seven genes prioritized by our resilience model significantly extended the lifespan of *C. elegans*. Given the important relationship between neuronal functional integrity and lifespan (i.e., lifespan vs. brain health span), this work suggests the predicted AD resilience genes could serve as important candidate targets for therapeutic intervention.

## 1. Introduction

Advances in medicine and public health measures, coupled with rising standards of living and improvements in education and nutrition have substantially increased human life expectancy. Since 1950, the life expectancy for the U.S. has increased by 16%, from 68 years to 78.9 years, and is projected to continue rising throughout this century [1]. A direct consequence of increased longevity is the rapid growth of the older population, accompanied by an increasing prevalence of aging-related diseases. In the United States, the elderly population (≥65) is projected to reach 83.7 million by 2050, nearly twice the estimated 43.1 million in 2012 [2]. It is estimated that the prevalence of aging related diseases (e.g., hypertension, coronary heart disease, heart failure, diabetes, Parkinson’s disease (PD), and Alzheimer’s Disease (AD)) in the elderly population of the U.S. will increase dramatically in the next decade [3]. Among these diseases, neurodegenerative cognitive disorders, including PD and AD, impose the greatest psychological, emotional, and financial burdens on the affected families and society [4]. The annual health care cost for AD patients are estimated to be between $159 billion and $215 billion in the United States and exceed $600 billion worldwide [5]. The number of people affected by AD is expected to increase by 250 percent between 2018 and 2050 (i.e., from 5.5 million to 13.8 million) [4].

AD is the most common form of dementia and accounts for an estimated 60 to 80 percent of late-life cognitive failure cases [4]. AD is an aging-related neurodegenerative disease of the brain characterized by progressive failure of all cortical function, culminating in death [6,7]. The underlying causes of typical late-onset sporadic AD (LOSAD) are still unknown, although the *APOE ɛ4* allele increases the risk of LOSAD dramatically [8].

Aging is the most crucial risk factor for the development of LOSAD. The prevalence of dementia, predominantly AD, rises rapidly after the age of 65 [7]. AD prevalence has been observed to be 15 times higher in 85-year-olds than in 60-year-olds [7]. Conversely, a significant proportion of the population remains cognitively intact despite being elderly (age > 85), and in the brains of some of these elderly individuals, there has been documentation of sufficient neuropathology to meet the criteria for pathological diagnosis of AD [9]. Markers of subjects who manifest “successful functional aging” (despite having accumulated a burden of cerebral pathology sufficient to merit a diagnosis of AD) include: (i) high cognitive and physical functioning, (ii) life satisfaction, (iii) longevity, (iv) freedom from disability, and (v) normal social function. [10]. High cognitive and physical functioning is probably the most essential feature of successful aging since robust mental and physical health are fundamental to other elements of successful aging. Understanding the molecular underpinnings of successful aging, especially those factors responsible for preservation of brain function, may provide novel insights as we seek to identify new interventions for prevention or treatment of aging-related neurodegenerative diseases, such as AD.

Previous studies have suggested that slowing aging or increasing lifespan, e.g., through genetic modifications, calorie restriction, or pharmacologic agents, may also postpone aging-related diseases [11]; therefore, targeting the aging process itself may be a convenient way to combat aging-related diseases [12]. The goal of the current study is to systematically identify molecular targets and pathways underlying successful aging by comparing the molecular mechanisms underlying normal aging and AD development. Specifically, we first explored the molecular mechanisms underlying normal aging with a gene expression dataset (GSE53890) from postmortem brain tissues collected from young and aged individuals who were cognitively intact with no evidence of neurological abnormalities [13]. By integrating the findings with the analyses on an AD cohort, we identified genes that are associated with aging and AD resilience, and in addition, the gene co-expression network structures that enabled the identification of key molecular drivers of successful aging in the brain. Our findings shed new light on molecular mechanisms underlying aging and AD resilience and thus will facilitate the development of novel therapeutics battling AD by targeting the key drivers involved in successful aging.

## 2. Materials and Methods

### 2.1. Data Collection and Pre-Processing

We downloaded the gene expression profile (GSE53890) from a human brain aging study, which includes 41 healthy brain samples covering a wide age range from 24 to 106 years old [13]. The disease status and the age range of the dataset are ideal for studying normal aging in human brains. The study profiled the gene expression of the prefrontal cortex (PFC) in postmortem brain tissues using microarray technology (Affymetrix Human Genome U133 Plus 2.0 Array). The samples included young adult cases without neurological abnormalities, aged subjects that did not carry a diagnosis of AD or other neurodegenerative disease and showed neuropathological findings within the normal range for age [13]. The data were normalized using Microarray Suite version 5.0 (MAS 5.0), and log2 transformed [13]. We collapsed the probes representing the same genes into those with the highest variances across the samples. We annotated the probes with the Affymetrix Human Genome U133 Plus 2.0 Array annotation data (chip hgu133plus2) from Bioconductor. We only kept the common genes between the microarray dataset and the ones in the RNAseq dataset from the Religious Orders Study and Memory and Aging Project (ROSMAP) study for cross-study comparison [14]. After data preprocessing and quality control, the final dataset was composed of 41 PFC samples covering 14,844 unique genes. Considering the risk for developing LOSAD is lowest under the age of 40 and highest in the population older than 85 [4], we divided the samples into three age groups: the young old (YO) group, the middle-old (MO) group, and the oldest old (OO) group (Table 1), to study the transcriptome changes across these populations.

This age stratification was also designed to align with the natural epidemiology of Alzheimer’s disease (AD), so that transcriptional signatures of brain aging could be interpreted against well-defined AD risk windows. Young Old (YO, 20–40) is a pre-risk reference group. Age 40 is a conservative lower bound for any AD onset—even autosomal-dominant early-onset AD driven by APP, PSEN1, or PSEN2 mutations very rarely manifests before the early 40 s, with the bulk of early-onset cases presenting between 45 and 64 [15]. Medium Old (MO, 41–84): the full at-risk window. Early-onset AD (EOAD) is defined as symptom onset before 65 and accounts for roughly 5–10% of cases, while late-onset AD (LOAD) prevalence rises steeply after 65. By 84, individuals have lived through essentially the entire period during which clinical AD is expected to emerge. Oldest Old (OO, 85+): The “oldest old” is an established demographic category in aging and dementia research [16]. Cognitively healthy individuals who reach 85+ have, by virtue of inclusion, escaped clinical AD despite traversing the highest-risk period—a phenomenon described in the literature as cognitive resilience or AD escape [17]. This group is therefore uniquely informative for identifying molecular signatures of resistance to neurodegeneration. In summary, the age stratification allowed us to contrast three biologically meaningful states—pre-risk, at-risk, and resilient—and to distinguish transcriptional features of normal brain aging from those specifically associated with escape from AD.

To compare the gene expression patterns between aging and AD development, we also downloaded preprocessed RNA-seq data from the ROSMAP study from the AMP-AD portal at Synapse [14]. The gene expression profiles from these two studies are both from the PFC region, making the two studies comparable. Genes with expression values of more than one fragment per kilobase of transcript per million mapped reads (FPKM) in at least 10% of the samples were selected and corrected for confounding factors, including batch, PMI, RIN score, and sex.

### 2.2. Differential Expression Analysis

To identify the differentially expressed genes (DEGs) among the three age groups, we carried out a linear model analysis using the Limma package (v3.36.2) in R with default settings. Considering the proportion of the cell composition may vary during brain aging, we estimated the proportion of six cell types, including astrocytes, endothelial cells, microglia, neurons, oligodendrocytes, and oligodendrocyte precursor cells, and adjusted them as covariates in a linear model using the R package BRETIGEA v1.0.3 [18]. However, including the estimated cell-type proportions as covariates in our model eliminated all transcriptomic differences between the age groups, which may not correctly reflect the molecular changes during human brain aging. Although neuron loss is commonly observed in many neurodegenerative diseases, we believe such dramatic changes in cell composition may not occur in successfully aged brains. As a result, our final model did not adjust for cell type composition. We utilized the Benjamini–Hochberg (BH) method to correct for multiple testing. Genes with an adjusted *p* < 0.1 and a fold change (FC) > 1.2 were considered significantly differentially expressed (DE) between conditions.

We also performed a DE analysis between AD and non-AD samples in the ROSMAP dataset. *p*-values were adjusted, and the significance levels were set using the same method mentioned above. An adjusted *p* < 0.4 was used to decide if a gene shows an up/down trend in AD samples. The liberal FDR < 0.4 cutoff for differentially expressed genes in AD was a deliberate choice: a less stringent cutoff for identifying AD signatures increases the robustness of the identified AD-resilient genes, which show divergent trajectories in AD-resilient aging and AD.

### 2.3. Trend Analysis

We performed Jonckheere’s trend test to determine if a gene showed a significant trend shift across the age continuum. The *p*-values were adjusted for multiple testing using the BH method. Genes with an adjusted *p* < 0.05 were considered to have significant trends across the age groups and were labeled differentially trended genes (DTGs).

### 2.4. Co-Expression Network Analysis for the Aging Data

We constructed gene co-expression networks for each age group to identify critical genes and gene networks associated with the age groups using the R package MEGENA (v1.3.7), which uses a multiscale clustering algorithm to identify modules for co-expressed genes and key drivers for each module from gene expression datasets [19]. We tested whether the DEGs and DTGs identified between age groups were enriched in the modules of each network. We then rank-ordered the modules in each network by the enrichment of the DEG/DTG signatures in each module. We also identified global key drivers for each network using key driver analysis [20]. The key driver genes of a network were then overlapped with DEGs and DTGs from the above analysis to identify the potential regulators of the aging/AD resilience genes.

### 2.5. Modular Differential Connectivity (MDC) Analysis

We performed the MDC analysis to detect and quantify the network reorganization between different age groups [21]. MDC compares the average connectivity (co-regulation of a gene pair) across all gene pairs within a module between age groups. MDC > 1 indicates a gain of connectivity (GOC) or enhanced co-regulation between genes, whereas MDC < 1 indicates a loss of connectivity (LOC) or a reduced co-regulation between genes.

### 2.6. Differential Gene Correlation Analysis

To further study the gene–gene interactions during aging, we examined the changes in correlations between individual gene pairs between age groups, i.e., OO vs. YO, OO vs. MO, and MO vs. YO. This analysis was performed using the R package DGCA (v1.0.1) with the default settings [22]. DGCA assesses nine types of changes in gene–gene correlation (+/+, +/0, +/−, 0/+, 0/0, 0/−, −/+, −/0, and −/−) between two conditions, in which “+” represents positive correlation, “−” represents negative correlation, and “0” represents no correlation. A “+/−” change represents a positive to a negative change in correlation between two conditions, and a “−/+” change represents a negative to positive change in correlation, etc. For each comparison, gene pairs with significantly altered correlation (FDR < 0.05) were extracted for the construction of a differential correlation network. Modules and key drivers in each differential correlation network were identified using the ddMEGENA function in the DGCA package v1.0.3.

### 2.7. Gene Set Enrichment Analysis

We carried out set enrichment analysis for gene sets such as DEGs, DTGs, cell-type-specific gene sets, and module genes using Fisher’s exact test [23]. The test assumes the gene sets were identically independently sampled from the genome-wide genes profiled. The *p*-values were adjusted for multiple testing using the BH method. We performed functional enrichment analyses for the above gene sets. The gene ontology (GO) annotations and canonical pathways (KEGG and Reactome) gene sets for the enrichment analysis were obtained from the Molecular Signatures Database (MSigDB) v6.1. We also performed edge set enrichment analysis (ESEA) using the R package ESEA (v1.0). We used FDR-adjusted *p* < 0.05 as the significance cutoff. Unlike the classical node-centric methods that use statistical models to detect whether a gene set is over- or under-represented in a predefined pathway, ESEA is an edge-centric method for functional enrichment analysis, which identifies dysregulated pathways by integrating pathway structure and differential correlation among genes [24].

### 2.8. C. elegans Protocols

N2 (wildtype, or WT) *C. elegans* were acquired from the Caenorhabditis Genetics Center (CGC) at the University of Minnesota. Maintenance procedures followed were similar to those previously described [25]. In brief, worms were maintained on Nematode Growth Medium (NGM) in the presence of OP50-1 bacteria, a strain of E. coli that is a uracil auxotroph and streptomycin-resistant, at 20 °C. RNAi was used to conditionally knock down the gene targets after the developmental stages of the worm. This was best accomplished using an RNAi feeding method previously described. The RNAi constructs used for feeding were acquired through Dharmacon, Horizon Inspired Cell Solutions (C. elegans RNAi v1.1 feeding library). The plasmid containing the RNAi constructs (pLL4440-dest) is ampicillin-resistant; this allows for the selection of bacteria containing the plasmid, increasing the effectiveness of knockdown. Three replicates for each condition were made. The plasmid containing no RNAi construct (empty vector or EV) served as the control for the experiment. N2 was used to perform an analysis of the effect on lifespan when knocking down the selected genes. To replicate the bioinformatic selection algorithm, when adult day 0 was reached, the worms were placed onto experimental plates with maintenance bacteria and allowed to grow until adult day 5, at which time they were then transferred to RNAi condition plates. Worms were assessed for survival by prodding them with a platinum wire. An additional RNAi control was added, daf-2, which has been shown to increase lifespan in C. elegans significantly [26]. The study was conducted under blinded conditions.

## 3. Results

### 3.1. Genes Involved in Nervous System Function Are Down-Regulated in Aged Brains While Those Involved in Immune System/Defense Response Are Up-Regulated

**Figure 1:**
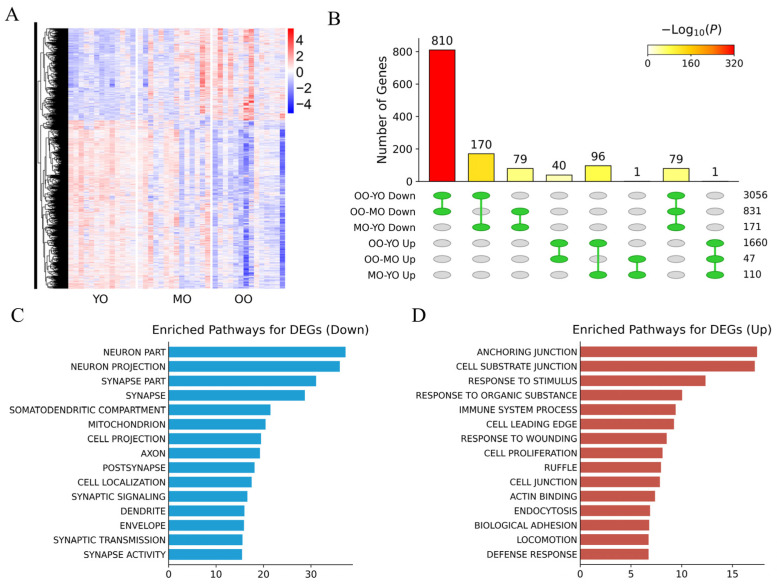
Intersections of DEGs identified between age groups and the functional enrichment for the DEG sets. (**A**). Expression of DEGs identified between age groups. Samples were categorized into three age groups–YO, MO and OO. (**B**). The matrix of solid and empty circles at the bottom illustrates the “presence” (solid green) or “absence” (empty) of the DEG sets in each intersection. The numbers to the right of the matrix are set sizes. The colored bars on the top of the matrix represent the overlap sizes, with the color intensity showing the *p*-value significance. (**C,D**). The top enriched functions/pathways for the DEGs identified between the OO and YO groups. The left panel (blue) shows the top enriched functions/pathways for the genes significantly down-regulated in the OO group (**C**), and the right panel (red) shows the top enriched functions/pathways for the genes significantly up-regulated in the OO group (**D**). *X* axes represent −log10(FDR).

We performed differential expression analysis between pairs of all age groups among YO, MO, and OO. We were primarily interested in the comparisons between OO and each of the other age groups, as OO has the highest risk of developing AD. The OO group is the fastest-growing segment of the population in the U.S. and is estimated to account for 12% of the population over 65 by 2050 [27]. At an FDR of <0.1 and FC > 1.2, we identified 4716 (OO vs. YO), 878 (OO vs. MO), and 281 (MO vs. YO) DEGs, respectively (Supplementary File S2). A large number of DEGs were identified between OO and YO; coupled with their distinct expression patterns, this suggests a substantial alteration in the transcriptome of the aged PFC compared to the young PFC (Figure 1A). In each comparison, there were more age-associated down-regulated DEGs than there were up-regulated DEGs. The overlaps between DEG signatures are shown in Figure 1B.

The most prominent differences were observed in the comparison between OO and YO. The genes downregulated in OO compared with YO were mainly involved in the modulation of synaptic transmission and mitochondrial function, and were associated with neurodegenerative diseases such as AD, PD, and Huntington’s disease (Figure 1C). In contrast, the genes upregulated in OO versus YO were associated with gap junction anchoring, immune function, and immune defense response (Figure 1C, Supplementary File S1). To investigate whether the aging process in the PFC was associated with particular cell types, we tested the enrichment of the DEG signatures for the marker gene signatures specific for astrocytes, endothelial cells, microglia, neurons, and oligodendrocytes [23]. The down-regulated genes in OO versus YO were enriched for neuronal genes, while the up-regulated genes were enriched for the marker genes of the other four brain cell types (Supplementary File S2). Similarly, the top pathways for genes down-regulated in OO compared with MO or genes down-regulated in MO compared with YO were related to synaptic and/or neuronal function. No significantly enriched pathways were observed for the genes up-regulated in OO compared with MO or genes up-regulated in MO compared with YO.

These results indicate that in the PFC, neuronal gene expression changes underlie the most prominent modification of the transcriptome of the aging brain. Another vital brain modification observed in the functional enrichment analysis is the activation of the immune system in the aged brain. Many studies have suggested the critical role of the immune system in regulating the progression of brain aging and neurodegenerative diseases [21,28].

### 3.2. Gene–Gene Interactions Were Significantly Different Between Age Groups

**Figure 2:**
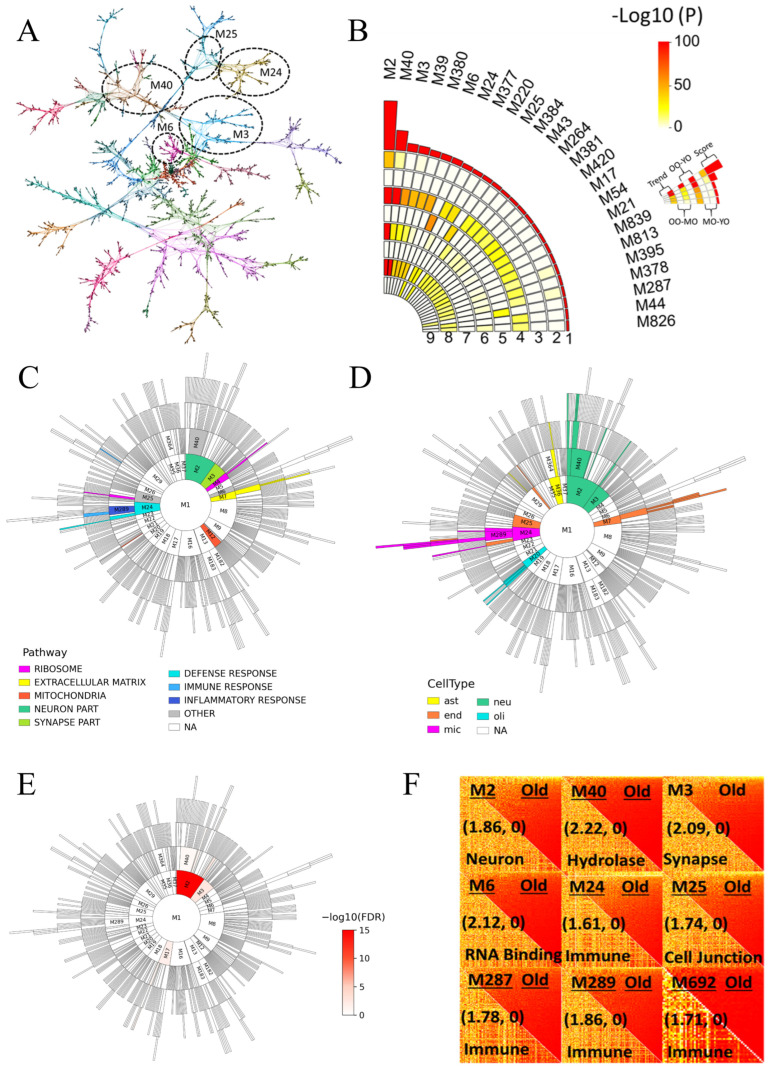
Gene co-expression network analysis of the OO group. (**A**). The global gene co-expression network for the OO group. Each color in the network represents a module. The top-ranked modules are highlighted and labeled. (**B**). The top-ranked modules. From outside to inside, the bar chart at track 1 shows ranking scores, the heat maps at tracks 2, 4, 6 and 8 show −log10 (*p* value) of the enrichment for the up-regulated DEGs/DTGs identified between/across age groups; the heat maps at tracks 3, 5, 7 and 9 show −log10 (*p* value) of the enrichment for the down-regulated DEGs/DTGs identified between/across age groups. (**C**–**E**). Major cell types, pathways, and AD resilience associated with the modules in the OO network. (**F**). Comparison of the network structure between the OO network and the YO network. Individual topological overlap matrices (TOM) of 9 differentially connected modules in the OO network (the upper right triangle of each module) versus those in the YO network (the lower left triangle of each module). Differential connectivity (MDC) and FDR estimates are specified in each panel in parentheses (MDC and FDR). Color intensity is proportional to topological overlap (TO): redder colors indicate higher TO values. The top enriched functions/pathways for each module are specified in each panel.

To gain more insights into the aging-associated transcriptome changes in the PFC, we constructed gene co-expression networks for the OO group using the Multiscale Embedded Gene Coexpression Network Analysis (MEGENA) [19] (Figure 2A). The modules in the constructed network were rank ordered by the enrichment for the DEG and DTG signatures identified above (Figure 2B). For each module in the network, we queried the MsigDB database for enriched functions and pathways (Figure 2C, Supplementary File S3). To determine the cell-type specificity of these modules, we tested how cell-type-specific gene signatures are enriched in each module (Figure 2D).

The top modules in the OO network were enriched for neuronal and/or synaptic function and for the immune/host defense response (Figure 2C, Supplementary File S3). Consistent with the prominent representation of neuronal and immune pathways, the top modules of the OO network were almost solely enriched for gene signatures specific to neurons or those specific to microglia (Figure 2D, Supplementary File S3). These results indicate that synaptic plasticity, mitochondrial function, and the immune system play key roles in the progression of brain aging. Consistently, compared to other cell types, neurons are most vulnerable to pathological aggregation of amyloid-β peptide (Aβ) and tau in AD [29]. The functional enrichment and the cell-type-enrichment analysis for the networks constructed from elderly people’s brains suggest that the activation of immune and inflammatory pathways, and the crosstalk between microglia and neurons are common cellular processes shared by both normal aging processes and the “pathological aging” processes often considered to underlie neurodegenerative diseases [23].

To detect and quantify the network reorganization across age groups, we performed modular differential connectivity (MDC) analysis [21]. Compared to the YO group, almost all modules (98%) in the network constructed in the OO group showed statistically significant (FDR-adjusted *p* < 0.05) connectivity changes, all of which exhibited GOC. Similar modular connectivity changes were observed when comparing OO to the MO group (all 94% modules showing significant MDC exhibited GOC at the old age group), indicating that gene interactions were significantly altered by age. Figure 2F illustrates a direct comparison of the topological overlap matrices between networks constructed with YO and OO groups.

We also tested whether the modules in the OO network are conserved in the YO network in terms of gene composition using Fisher’s exact test. We found 60 modules in the OO network that significantly overlapped with 37 modules in the YO network, half of which are top ranked. The top-ranked modules showing MDC are also significantly conserved between OO and YO networks, suggesting the gene–gene interaction is an important component of the transcriptome alterations during aging.

### 3.3. Validation of Gene Co-Expression Network Using Perturbation Signatures

To evaluate whether the constructed co-regulatory network captured biologically meaningful co-regulation signals rather than random noise, we examined whether the gene–gene interactions in the network can also be detected in the gene perturbation experiments. In other words, we examined whether the predicted co-regulation of a specific gene and its neighbor genes in the network can also be observed in the perturbation experiment of this specific gene. For example, we found genes co-regulated with *TYROBP* in the co-expression network that significantly overlapped with the gene signature identified in a mouse *TYROBP* knockdown experiment (Table 2). The overlap indicates that the prediction of the co-regulation of *TYROBP* and its neighbor genes was accurate and validates our predictions as reflective of real biological events that occur in the living brain.

We collected a total of 13 gene perturbation signatures from the literature, which include gene knockdown/knockin signatures, gene overexpression signatures, and ChIP-sequencing signatures [13,21,30,31]. For all networks, we observed significant overlaps (*p* < 0.05) between the neighbors of the perturbed genes within five layers of the network and the corresponding gene signatures of the disrupted genes in almost all cases (Table 2), indicating the co-expression network we constructed was able to capture real gene interactions. However, not all the perturbation signatures are enriched in the respective subnetworks, and they include REST and YAP1. The REST signature was identified through ChIP-seq analysis of SH-SY5Y cells, while the YAP1 signature was derived from RNA-sequencing data generated from U251-APP cells. As both signatures were obtained from immortalized cell lines, their relevance to the complex biological processes underlying human longevity may be limited.

### 3.4. Successful Aging in the Face of Accumulating Brain Pathology as the Result of Orchestration of Various Neuroprotective Signaling Pathways

**Figure 3:**
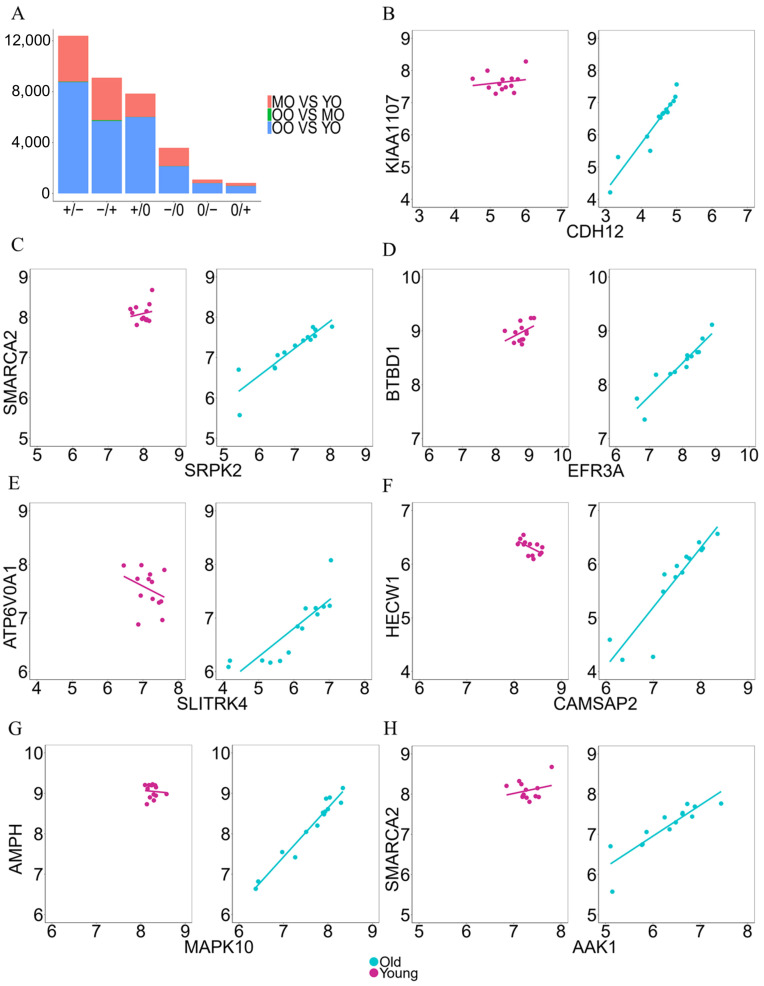
Differential correlation analysis between age groups. (**A**). The numbers and types of differential correlations identified in the three comparisons, i.e., MO vs. YO, OO vs. MO, and OO vs. YO. “+” represents positive correlation, “−” represents negative correlation, and “0” represents no correlation. A “+/−” change represents a positive to a negative change in correlation between two conditions, and a “−/+” change represents a negative to positive change in correlation. (**B**–**H**). The most significant correlation changes in the potential AD-resilient genes with other genes between the OO group and the YO group.

While co-expression network analysis is useful in clustering similarly expressed genes to derive a global view of the gene–gene coregulation structures, it does not reveal interaction changes at a resolution of individual gene pairs. To scrutinize changes in gene–gene interactions between any two age groups, we performed differential gene correlation analysis (DGCA). The numbers and types of correlation changes for each comparison are shown in Figure 3A. YO and OO groups had the largest number of correlation changes. Figure 3B–H shows examples of the correlation changes between YO and OO from a list of our candidate genes involved in AD resilience. Among the three comparisons (OO vs. YO, OO vs. MO, and MO vs. YO), most differential correlations were identified between OO and YO. The most abundant differential correlation types identified between OO and YO were “+/−”, “−/+”, and “+/0”. The top enriched pathways for the genes in the three major differential correlation types were dominated by genes for neuronal and/or synaptic functions (Supplementary File S4), suggesting that a drastic co-regulation change among genes is involved in maintaining normal cognitive functions during brain aging and presumably during the accumulation of AD pathology.

To complement the node-centric pathway enrichment analysis, which tests if a set of genes is over- or under-represented in a predefined pathway, we carried out an edge-centric gene set enrichment analysis (ESEA). Although both DGCA and ESEA try to identify the shift in pathways by investigating the changes in inherent biological relationships between genes, the two methods utilize substantially different strategies. DGCA identifies differentially correlated gene pairs first and then examines the biological pathways of the enriched genes for each correlation change category. In contrast, ESEA first establishes a global gene interaction network based on the pathway databases and then maps the gene expression data to this network [24]. In this study, we used both algorithms to improve our understanding of the biological relationships between genes embedded in the remodeled pathways during brain aging.

In ESEA, the correlation between the expression profiles of two genes is estimated using mutual information (MI). The differential correlation score for an edge (*EdgeScore*) is the difference in the MIs between two age groups. During comparisons, gene pairs are classified as representative of either a gain of correlation (GoC), a loss of correlation (LoC), or no change in correlation (NC), with the EdgeScore greater than, less than, or equal to zero. We performed ESEA on three comparisons: OO vs. YO, OO vs. MO, and MO vs. YO. The common pathways identified for the gene pairs showing GoC across the three comparisons include tubulin folding, 5HT2 type receptor-mediated signaling pathway, oxidative phosphorylation, Nef-mediated CD4 down-regulation, retrograde endocannabinoid signaling, and WNT signaling (Supplementary File S5). The common pathways for the gene pairs showing LoC underlie the formation of the beta-catenin-TCF transactivating complex, as well as PRC2 methylated histones and DNA (Supplementary File S5). Oxidative phosphorylation is an essential mechanism that provides energy to power neuronal activities in normal brain function [32]. The *WNT* signaling pathway has been found to be crucial to normal brain function, as dysregulation of this pathway is associated with mental disorders, mood disorders, and neurodegenerative diseases [33]. Most interestingly, a proper regulation of the *WNT* signaling pathway has been demonstrated to be neuroprotective in AD [34]. CD4 is a glycoprotein found on the surface of immune cells, which may contribute to neuronal damage in infectious and immune-mediated diseases of the central nervous system [34]. The serotonin 5-HT2 pathway is involved in learning and memory, spatial cognition, and mental disorders [35]. The chaperonin TRiC/CCT facilitates the folding of many proteins, including cytoskeletal components and cell cycle regulators [36]. Retrograde endocannabinoid signaling regulates axonal growth and guidance during development and drives adult neurogenesis throughout the brain [37]. Consistent with the DGCA, the ESEA suggests that pathways involved in normal brain functioning during brain aging are regulated by the dynamic changes in a host of gene–gene interactions and their co-regulations.

### 3.5. Identification of Key Driver Genes Involved in AD Resilience

To further capture the patterns of the changing transcriptome during brain aging, we examined the expression trend of each gene across the three age groups using Jonckheere’s trend analysis. We found 4124 genes that showed significant expression trends across the age groups, among which 2763 genes showed a trend of decreased expression, and 1361 genes showed a trend of increased expression (Supplementary File S6). Similar to DEGs, DTGs with downtrends were significantly enriched for nervous system function, mitochondrion, and neurodegenerative diseases. In contrast, genes with uptrends were enriched for cell junctions and immune/defense response (Supplementary File S6).

To identify genes that are potentially “aging-protective” and that show differentially altered expression patterns between age groups, we integrated the results of the trend and differential expression analyses. The DGCA and ESEA analyses suggest that pathways involved in normal cognitive functioning during brain aging are regulated by drastic changes in the gene co-regulation network. We therefore hypothesized that genes responsible for these substantial changes may themselves undergo extreme expression changes during brain aging. Based on our hypothesis, we examined three scenarios to address the gene expression changes during aging (Supplementary File S1). Genes showing expression patterns in any one of these described scenarios were deemed as aging-protective genes. In the first scenario, a gene shows an upward trend from YO to MO but loses this trend from MO to OO. The second scenario includes genes with a downward trend from YO to MO but lose this trend from MO to OO. In the third scenario, a gene has no expression trend changes from YO to MO but displays either an upward or a downward trend from MO to OO. We did not detect genes with expression patterns matching the first or second scenario. However, we identified 798 genes fitting the third scenario. These 798 genes are a subset of the DEGs identified between OO and MO, in which genes downregulated in the OO group are mainly enriched for compensatory mechanisms that sustain nervous system functioning. No significantly enriched pathways were observed for the up-regulated genes.

**Figure 4:**
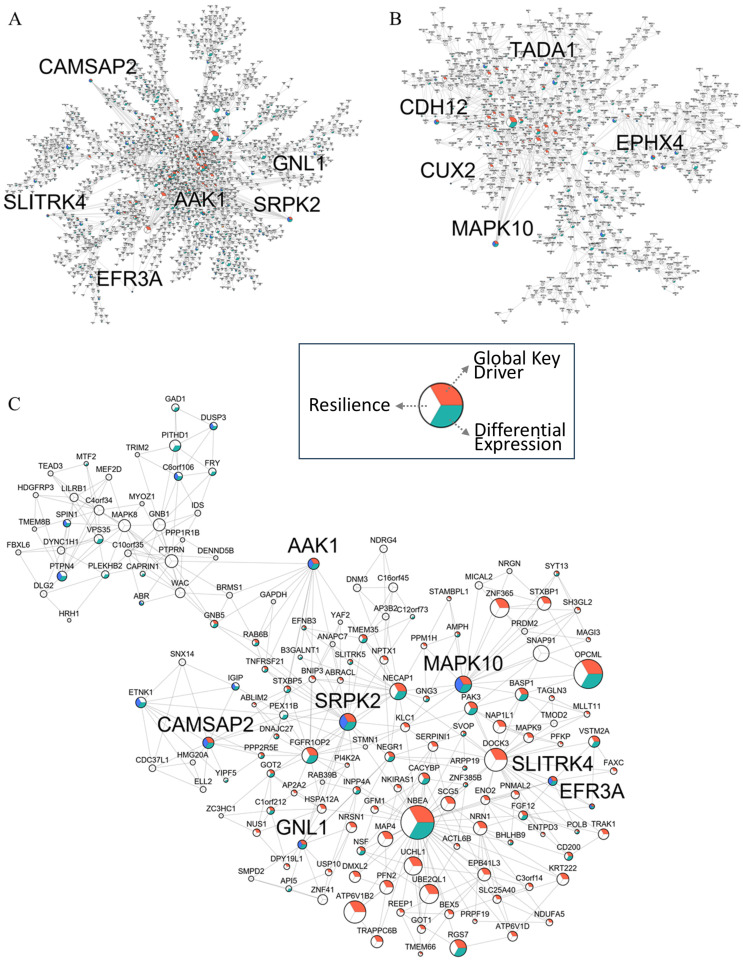
The top-ranked module and the subnetworks centered around the top-ranked AD-resilience genes in the OO network. (**A**,**B**). The top-ranked module M2 (**A**) and M3 (**B**) in the OO network. The top AD-resilience genes in the module M2 are highlighted with a large font size. The size of a node is proportional to the node connectivity in the co-expression network. In the pie chart on each node, the upper-right sector indicates whether the corresponding gene is a global key driver (GKD) whose neighbors are enriched for AD-resilience genes, with the red fill-in color representing a GKD and the white fill-in color representing a non-GKD. The left sector of each pie chart indicates whether the corresponding gene is an AD-resilience gene, with the blue fill-in color representing an AD-resilience gene and the white fill-in color representing a non-AD-resilience gene. The lower-right sector of each pie chart indicates whether the corresponding gene is differentially expressed between the OO and MO groups, with the orange, green, and white fill-in colors representing upregulation, downregulation, and no differential expression, respectively. (**C**). The subnetworks centered around the top-ranked AD-resilience genes (labels with large font size) in the OO network.

To pinpoint the key regulators that potentially drive AD resilience, we examined the “aging-protective genes” identified above and the DEGs identified between the AD and non-AD groups in the ROSMAP dataset [38]. We developed three criteria to identify AD-resilient genes amongst the aging-protective genes. We hypothesized that genes involved in AD resilience are aging-protective genes that show differential expression patterns between successful aging and AD development. Therefore, an AD-resilient gene must meet one of the following criteria. (1) The gene is an aging-protective gene but is not differentially expressed between AD and control. (2) The gene is an aging-protective gene and is differentially expressed between AD and control, but its expression moves in opposite directions between the two populations (OO and AD). (3) The gene is a network key driver in the OO network, and its neighbors (4-layer) in the network are enriched for the genes that meet the criteria #1 or #2. We found that 282 genes met at least one of the three criteria (Supplementary File S7), which significantly overlapped the top modules of the OO network (Figure 2E). Among these genes, eleven genes met the third criterion: *AAK1*, *CAMSAP2*, *GNL1*, *CUX2*, *EFR3A*, *SRPK2*, *CDH12*, *TADA1*, *MAPK10*, *SLITRK4*, and *EPHX4*. All the eleven genes are members of the top modules (enriched for neuronal and/or synaptic function) of the OO network (Figure 4A,B). In addition, over a half of these candidate genes are closely connected in the OO network (Figure 4C), suggesting that these genes are orchestrated to preserve normal cognitive functions in the brain.

### 3.6. AD Resilience Genes Identified in the Human Brain Extended the Lifespan of C. elegans

Neuronal activity is a major determinant of lifespan [39]. Early evidence of this phenomenon was that inhibition of sensory neuronal activity increases lifespan in *C. elegans* [40,41] and *Drosophila* [42]. Similarly, just two sensory neurons mediate the life-extending effects of dietary restriction in *C. elegans* [43]. Inhibition of neuronal genes regulating neurosecretion also increases lifespan in *C. elegans* [44]. Furthermore, neurons mediate the increase in lifespan produced by inhibiting insulin-like signaling [45], which increases lifespan in *C. elegans* [26], *Drosophila* [46], mice [47], and in humans delays aging-related diseases [48]. Brain expression of the transcriptional co-activator Creb-binding factor (CBP) correlates positively with lifespan in mice and mediates the life-extending effects of dietary restriction and inhibiting insulin-like signaling [25], effects that are mediated by GABA-ergic neurons [49]. Conversely, inhibition of the transcriptional co-inhibitor CtBP, which inhibits CBP activity [50], increases lifespan [51], also an effect mediated by neurons [52]. This observation is particularly interesting in view of evidence that longevity in humans is associated with reduced expression of excitatory genes in the cerebral cortex, associated with increased activity of the inhibitor transcription factor REST/NRSF, and inhibition of neuronal activity increases lifespan in *C. elegans* and *Drosophila* [53]. REST/NRSF mediates the anti-epileptic effect of glycolysis inhibition metabolism by recruiting CtBP [54]. Therefore, it is of great interest to see if the candidate AD resilience genes, which were predicted to be involved in normal cognitive function, are also able to mediate lifespan variation in *C. elegans*.

**Figure 5:**
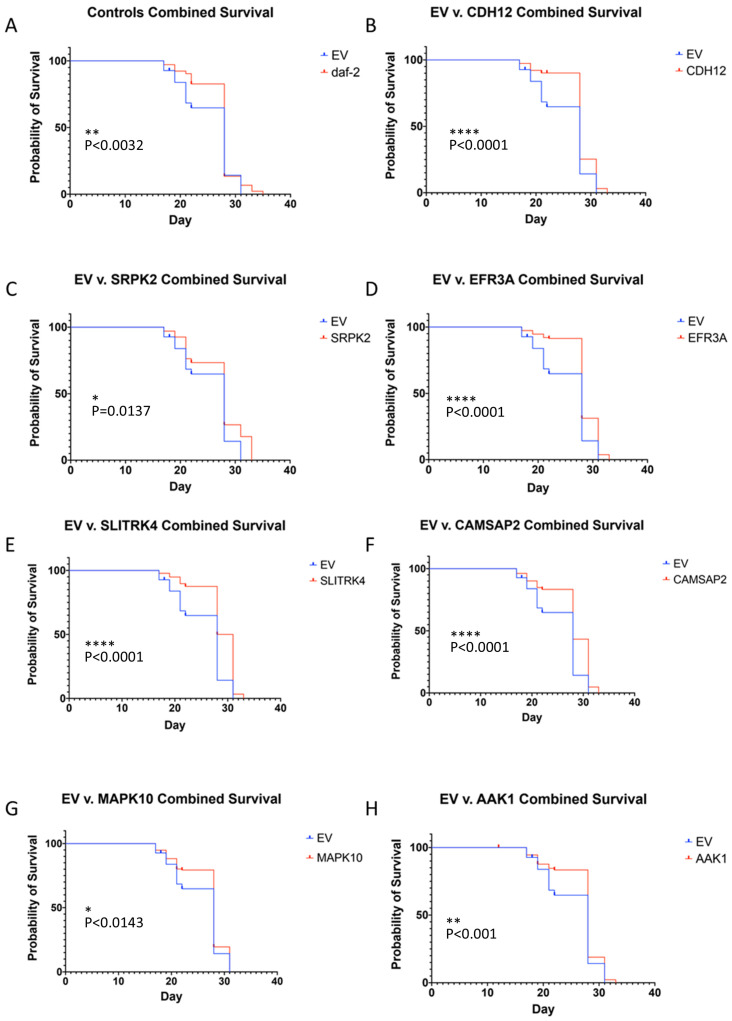
RNAi inhibition of AD-resilience genes significantly extends *C. elegans* lifespan. Statistical significance (*p* < 0.05). (**A**–**H**): Effects of aging-protective genes inhibition on *C. elegans* lifespan. “Combined Survival” indicates that the results of two separate experiments were combined. The first experiment ended when the control (EV) reached 50% survival probability, whereas the second experiment ended when the last worm was marked as dead in all the conditions.

Among the eleven candidate genes previously identified as potential regulators of AD resilience, seven possessed known C. elegans homologs and thus were consequently prioritized for experimental testing in C. elegans, including *CDH12*, *EFR3A*, *SRPK2*, *SLITRK4*, *CAMSAP2*, *MAPK10*, and *AAK1*. Since all seven genes were specifically down-regulated in the successfully aged OO group and negatively correlated with age (Supplementary File S2), we predicted that knocking down these genes may extend the lifespan of wild type *C. elegans*. Since inhibition of daf-2 expression extends *C. elegans* lifespan [55], daf-2 inhibition was used as a positive control in the gene knockdown experiments. We performed two independent RNAi experiments. The first experiment concluded when the control (EV) reached 50% survival probability, while the second experiment ended when the last worm was marked as dead in all the conditions. The results of the two experiments were combined for statistical analysis. For all tested AD resilience genes, we observed significantly increased lifespan in the worms when the gene was knocked down (*p* < 0.05, Figure 5B–H). These results indicate that longevity and aging-related neurodegeneration are intertwined at the molecular level and that disrupting the molecular network underlying one process will also significantly change the alternative process.

### 3.7. Effects of AAK1 Inhibitors on Oligomeric Abeta (oAβ) Toxicity and Spontaneous Neuronal Activity in Primary Murine Brain Neuron–Glial Cultures

Next, we explored whether AAK1 inhibition may modulate neurodegeneration. According to the Human Protein Atlas (https://www.proteinatlas.org/ENSG00000115977-AAK1/single+cell+type, accessed on 15 January 2020), in the brain, AAK-1 is highly expressed in neurons. Based on this, we explore whether AAK1 inhibition may modulate Aβ-mediated toxicity in primary murine brain neuron–glial co-cultures. To this end, we selected two potent AAK-1 inhibitors (LP-935509 and LP-922761) identified by Kostich et al. [56] for our in vitro study.

**Figure 6:**
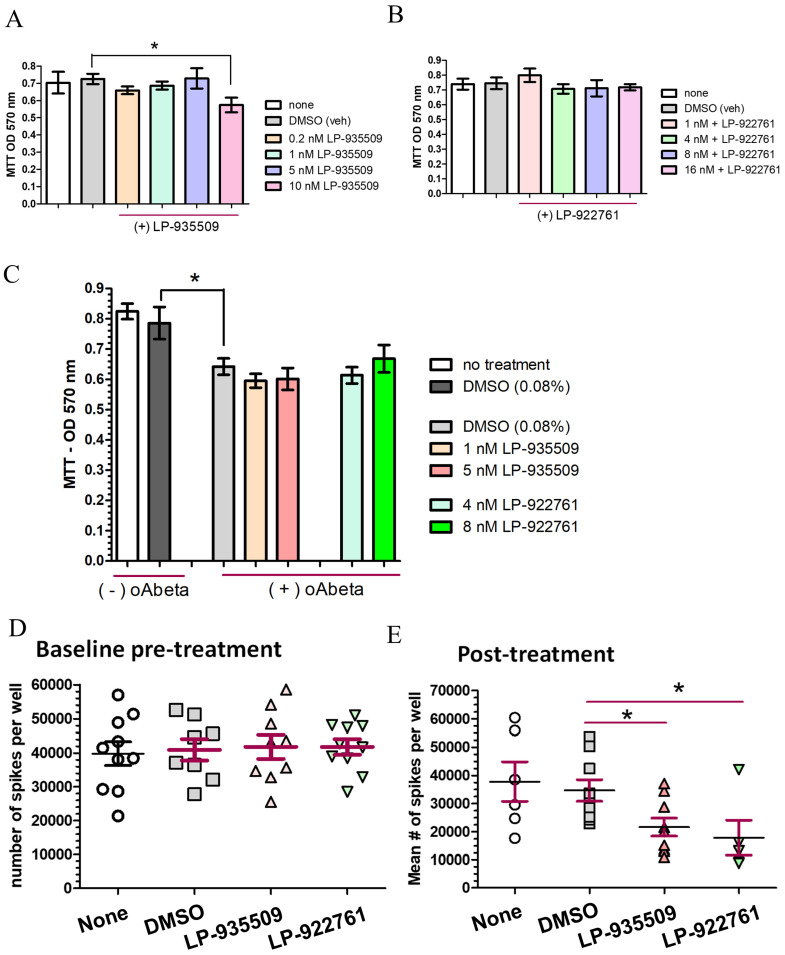
Effects of AAK-1 inhibitors on oligomeric Abeta (oAβ) toxicity in primary murine brain neuron–glial cultures. (**A**,**B**) Dose-responsive tolerability of AAK-1 inhibitors in primary murine neuron–glial co-cultures. Matured co-cultures (DIV 18) were treated for 24 h with increasing doses of LP-935509 (**A**) or LP-922761 (**B**). Cell viability was then assessed using the MTT assay. Bar graphs depict mean +/−se; = 7–8. For LP-935509 treatments. 1-way ANOVA *p* = 0.1458; a significant difference was observed comparing DMSO to 10 nM LP-385509 treatments (*t*-test *p* = 0.013), while for the LP-922761 treatments, 1-way ANOVA *p* = 0.6147; no significant difference was observed comparing DMSO to any of the LP-385509 treatments. (**C**) Effects of AAK-1 inhibitors on oAβ-mediated toxicity in matured primary murine neuron–glial co-cultures. Matured co-cultures (DIV18) were treated with oAβ (5 μM) in the absence or in the presence of oAβ-treatment with varying doses of LP-935509 or KP-922761 (as indicated) for 24 h. Cell toxicity was monitored using the MT assay. Bar graphs depict mean +/−se; n = 7–8. 1-way ANOVA *p* < 0.0001; * post-hoc Bonferroni’s Multiple Comparison Test: DMSO vs. oAβ, *p* < 0.05. No significant difference was observed comparing oAβ + DMSO vs. oAβ + 1 or 5 nM LP-385509 or oAβ + DMSO vs. oAβ + 4 or 9 nM LP-922761. (**D**,**E**) Effects of AAK-1 inhibitors on spontaneous neuronal activity in matured primary murine neuron–glial co-cultures. Scatter graphs showed spontaneous neuronal activity presented as the mean number of neuronal spikes per well (n = 8–10 wells per experimental group). (**D**) Basal spontaneous activity monitored at DIV18. 1-way ANOVA *p* = 0.9623. (**E**) Spontaneous neuronal activities 24 h after vehicle or drug treatment. 1-way ANOVA *p* = 0.026.

Based on the identification and characterization of multiple AAK-1 inhibiting compounds reported by Kostich et al. [56], we selected two potent AAK-1 inhibitors, LP-935509 and LP-922761, for our in vitro study. The reported IC50 value of LP-935509 for AAK-1 inhibition is 3.3 nM. However, at significantly higher concentrations, LP-935509 could also inhibit BIKE (BMP-2-inducible protein kinase) and GAK (cyclin G-associated kinase) (the IC50 values for BIKE and GAK are 12 nM and 320 nM, respectively) [56]. The reported IC50 value for LP-922761 is 7.6 nM. LP-922761 can also inhibit BIKE at significantly higher concentrations (IC50 = 24 nM), but the compound does not inhibit GAK [56]. We evaluated the dose-dependent tolerability of AAK-1 inhibitor treatment in primary neuron–glial co-cultures. For LP-935509, we observed no significant toxicity across drug concentrations ranging from 0.2 to 5 nM. However, observable toxicity was detected at a drug concentration of 10 nM (Figure 6A). No cellular toxicity was observed for LP-922761 across drug concentrations ranging from 1 to 16 nM (Figure 6B). We next evaluated the effects of the two AAK-1 inhibitors on oligomeric Aβ (oAβ)-mediated toxicity. As shown in Figure 6C, treatment with 5 μM oAβ induced significant toxicity in primary neuron–glial co-cultures. There is no observable change in oAβ toxicity in response to co-treatment with 1 or 5 nM LP-935509 or with 4 or 8 nM LP-022761 (Figure 6C).

Spontaneous neuronal activity, defined as non-evoked or stimulus-independent activity, is a fundamental property of nervous systems [57]. Such spontaneous firing plays a central role in transforming synaptic input into spike output and encoding plasticity in neural circuits [58]. In a rat model of chronic constriction injury, inhibition of AAK-1 in the spinal cord was shown to attenuate the increased spontaneous neuronal activity induced by the chronic constriction injury [56]. Based on this previous finding, we sought to explore whether AAK-1 inhibition might similarly modulate spontaneous neuronal activity in brain neurons. We treated mature neuron–glial co-cultures with 3 nM LP-935509 or 8 nM LP-922761, doses close to the reported IC_50_ values for these AAK-1 inhibitors and well tolerated by primary murine brain neuron–glial co-cultures (Figure 6D,E). We observed that both drug treatment regimens significantly inhibited spontaneous neuronal activity (Figure 6D,E). In the view that an impairment of synaptic plasticity mechanisms may play a key role in AD pathogenesis, our evidence suggests AAK-1 as a target for promoting synaptic plasticity in the AD brain.

## 4. Discussion

AD is an age-related, progressive neurodegenerative disorder characterized by the accumulation of intracellular and extracellular protein aggregates, accompanied by neuronal and synaptic loss, and frequently (though not invariably) resulting in clinically significant cognitive impairment and, ultimately, death [59]. Although aging has been considered the most significant risk factor for AD, a substantial proportion of individuals aged over 85 years continue to live with preserved cognitive function and a quality of life until their terminal illness and death. In this study, we examined gene expression patterns in the human PFC of 41 individuals with ages ranging from 24 to 106, who did not carry a diagnosis of AD or any other neurodegenerative disease.

Based on the risks for developing LOSAD at different ages, we classified these individuals into three age groups (YO, MO, and OO) and compared gene expression patterns, trends, and connectivity across the three groups. We first explored the gene expression differences across the three age groups by performing DE analysis and trend analysis. Functional enrichment analyses indicate genes showing a decreased expression trend as the age increased are involved in synapse/neuron functions, while those showing an increased expression trend are implicated in the immune/defense system. These pathways were also observed in many AD studies, in which genes down-regulated in AD are enriched in synapse/neuron pathways while those up-regulated in AD are involved in the immune response [60,61,62,63], suggesting common molecular underpinnings of normal brain aging and AD. We also examined the changes in gene–gene interactions across the age groups as well as the pathways in which they are involved using DGCA and ESEA, confirming that neuronal and/or synaptic function and immune system activation are major biological events during brain aging.

We were most interested in the OO group in which aged individuals (85+ years old) maintained normal cognition. To identify genes that are responsible for successful aging, we adopted a strategy illustrated in Supplementary File S1. Briefly, we hypothesized that aging resilience genes would show striking changes, i.e., opposite expression patterns, across age groups. We set three possible expression patterns as defining features for any potential aging-resilience gene (Supplementary File S1). With DE analysis and trend analysis, we were able to identify 798 aging-resilience genes showing the third of our three proposed expression patterns (Supplementary File S1C). Our strategy of identifying aging resilience genes has its limitations. Our process ignores genes showing consistent expression patterns across all age groups, and it is conceivable that these genes may nonetheless contribute to successful aging. However, our strategy was able to capture the aging-resilience gene candidates showing the most striking expression changes. We also constructed a gene co-expression network for each age group and compared the structures between the networks.

To identify potential AD-resilience genes, we compared the expression patterns of the aging resilience genes identified above and the DEGs identified between AD and control from the ROSMAP study. Considering the close relationship between AD and aging, we hypothesized that AD-resilience genes are aging-resilience genes that show significant expression differences between the resilience population (OO group in this case) and the AD population. Based on this hypothesis, we set up three criteria to identify AD resilience genes as we described in Section 3. We selected all eleven genes that met the third criterion—the most stringent criterion of the three—for further analysis.

Many of these predicted AD-resilience genes have been demonstrated to be involved in AD pathogenesis and memory. For example, *SRPK2* was reported to be downregulated in the resilience population (the OO group) in this study, suggesting a negative correlation between the expression of *SRPK2* and maintenance of normal cognition. SRPK2 is a protein kinase specific for the serine/arginine (SR) family of splicing factors, which mediates cell cycle progression and cell death in neurons in the brain [64]. As we predicted, deleting *SRPK2* alleviated the memory defect and improved the induction of long-term potentiation in APP/PS1 mice [65] while overexpression of *SRPK2* in 3×Tg mice significantly aggravated the cognitive impairment, while inhibition of SRPK2 attenuates AD pathologies and reversed cognitive defects [66]. Similarly, downregulation of *AAK1*, *EFR3A* and *MAPK10*, which were down-regulated in the OO group, has also been shown to attenuate AD pathogenesis [67,68,69]. These studies suggest that the AD-resilience genes we identified in this study are highly relevant, and the bioinformatics approaches we used to identify these genes are effective.

REST has been demonstrated to be closely correlated with cognitive preservation and longevity [13]. The activation of REST is necessary to protect neurons from oxidative stress and Aβ toxicity, while conditional deletion of REST in the mouse brain leads to aging-related neurodegeneration [13]. As REST showed a consistent trend of increased expression across the three age groups, the strategy used to identify aging-protective and AD-resilience gene candidates in this work was not able to locate REST as either one of the candidates. However, we found that the AD-resilience gene candidates significantly overlapped with the targets of REST (Fisher’s exact test, *p* < 0.0001), which were identified by ChIP-seq [13]. Additionally, we found SRPK2 (one of our top AD-resilience gene candidates) is a direct target of REST. More interestingly, we observed opposite expression trends between SRPK2 and REST across the various age groups. Given the current knowledge of neuroprotection and expression patterns of SRPK2 and REST in the aging brain, we believe SRPK2 suppression by REST is crucial to successful brain aging. These results indicate that a significant portion of the AD-resilience gene candidates identified in this work is involved in the REST-centered regulatory network underlying neuroprotection during brain aging.

Many studies have shown that delaying the onset of aging-related diseases, such as neurodegenerative diseases, can be associated with an extended lifespan. For example, lifespan extension via growth factor receptor, insulin receptor, and mTOR pathway inhibition has been shown to restrict aging-related neurodegeneration in various species and mediate Aβ deposition in the 3×Tg-AD mouse model [70]. Anti-aging manipulations have the potential to prevent AD and increase lifespan via the retardation of the aging process, which may be a feasible treatment of AD [71]. In a high-throughput screen for drugs that increase lifespan in *C. elegans*, anti-epileptic compounds (including the most protective such compound, ethosuximide) were shown to increase lifespan [72]. In a separate, high-throughput screen (88,000 compounds) for compounds that increase lifespan, one of the most robust was the antidepressant drug mianserin, which apparently increases lifespan by inhibiting neuronal serotonin transmission, possibly mimicking the effects of dietary restriction [73]. Subsequent analysis of the results of this screen clearly indicated that the vast majority of drugs that increase lifespan have some action on neurons [74]. A similar high-throughput screen also yielded a robust set of related compounds that increase lifespan through neuronal activity, apparently by masking satiety signals, thus leading to a perceived nutritional deficit [75]. There are many examples in which manipulations that protect in mammalian models of Alzheimer’s also increase lifespan in C. elegans [76,77,78,79,80,81,82], and the converse is also true (e.g., dietary restriction) [25]. To our knowledge, there have been no exceptions—and of course, increasing lifespan is a rare property compared to reducing lifespan. We therefore suggest that evidence that a manipulation increases lifespan may be considered as supportive that the same manipulation may protect against AD. Taken together, genetic and pharmacological evidence strongly indicates that neuronal activity plays a dominant role in determining lifespan. These observations suggest that cortical gene expression conferring cognitive resilience may plausibly also promote resilience in lifespan, although whether such genes are compelling targets for AD resilience remains to be further demonstrated.

In this study, we integrated two datasets generated from two different studies and two different profiling platforms. However, it is important to clarify that we did not directly merge the raw datasets into a single analysis. Instead, we analyzed each dataset separately and subsequently integrated the results. This strategy substantially reduces the potential impact of platform-specific biases, cohort heterogeneity, and other study-specific confounding factors, and thus enhances the robustness and reliability of the identified signals while minimizing technical artifacts that may arise from direct dataset combination.

One limitation of the present study is the use of bulk RNA-seq to identify resilience-related gene networks and signatures. While bulk RNA-seq provides valuable insights into global transcriptional changes associated with resilience against AD, it lacks the cellular resolution needed to distinguish molecular alterations occurring in specific brain cell populations. The observed neuronal downregulation and immune upregulation could be confounded by age-related shifts in cellular composition rather than true regulatory changes within cells. While adjustment for computationally estimated cell-type proportions attenuated many age-associated expression changes, such correction may also remove biologically meaningful aging signals because the proportions were inferred from the transcriptomic data itself and may reflect both cellular abundance and age-related transcriptional alterations. Emerging single-cell and single-nucleus RNA-seq approaches will enable a more precise characterization of cell-type-specific transcriptional programs underlying resilience by resolving heterogeneous cellular responses across neurons, astrocytes, microglia, oligodendrocytes, and vascular cells. These technologies can identify rare or selectively vulnerable cell populations, uncover protective signaling pathways that may be masked in bulk tissue analyses, and distinguish whether observed molecular changes reflect shifts in cell composition or true transcriptional regulation within individual cell types. In particular, single-cell studies may reveal resilience-associated microglial or astrocytic states, neuron subtype-specific stress responses, and cell–cell communication networks that contribute to preserved cognitive function despite AD pathology. Integrating single-cell transcriptomics with spatial and epigenomic profiling will therefore be essential for identifying actionable therapeutic targets and refining the mechanistic understanding of resilience against AD.

Another limitation of the study is the relatively small sample size in the discovery cohort. However, by applying advanced network biology methodologies, we identified molecular networks and key regulatory genes potentially involved in AD resilience. Notably, these findings were partially validated through independent in silico validation and in vivo experiments, supporting the robustness and biological relevance of the identified mechanisms despite the limited sample size. An important future direction will be to establish larger cohorts of individuals resilient to AD, which will provide greater statistical power for identifying the molecular and genetic mechanisms underlying AD resilience.

## 5. Conclusions

In this project, we systematically analyzed gene expression data from the prefrontal cortices of cognitively normal individuals to identify candidate aging-protective genes. Differential expression and gene co-expression network analysis identified key genes that may promote brain resilience and delay dementia. The prioritized key candidate genes with homologues in *Caenorhabditis elegans* were shown to extend the lifespan of *C. elegans*, supporting their potential roles in maintaining brain health and highlighting them as promising therapeutic targets for AD. The key findings, including the aging signatures, the gene network models, and the validated key aging-protective genes, shed new light on molecular mechanisms underlying aging and AD resilience and will facilitate the discovery of novel molecular mechanisms in successful aging and the development of novel therapeutics for AD.

## Figures and Tables

**Table 1:** The demographics of the gene expression data (GSE53890), divided into three age groups.

Group	Age (Range, Mean ± SD)	Tissue	Diagnosis	N (Female)	N (Male)	N (Total)
Young Old (YO)	20~40, 30 ± 7	PFC	Normal	7	6	13
Medium Old (MO)	41~84, 63 ± 15	PFC	Normal	6	8	14
Oldest Old (OO)	85+, 94 ± 7	PFC	Normal	8	6	14
Total	24~106, 63 ± 28	PFC	Normal	21	20	41

**Table 2:** Overlaps between the neighbors of the perturbed genes in the OO network and their perturbed signatures (Fisher’s exact test).

Gene	No. Neighbors	Signature Size	Fold Enrichment	FDR
PLP1	353	1111	3.1	4.03 × 10^−9^
LIPA	299	1995	2.4	3.21 × 10^−7^
TYROBP	418	1167	2.4	9.75 × 10^−7^
PSEN1	303	2609	2.3	1.56 × 10^−7^
SYPL1	306	1679	2	2.60 × 10^−4^
CNP	226	1261	1.9	1. 07 × 10^−2^
UGT8	375	2535	1.7	4.80 × 10^−4^
MAP7	152	2050	1.7	2.08 × 10^−2^
NPC1	274	4795	1.7	2.10 × 10^−4^
ERBB3	226	3256	1.5	1.44 × 10^−2^
REST	135	2792	1.1	0.44
SLC25A13	146	2328	1	0.56
YAP1	583	430	0.6	0.92

## Data Availability

The microarray gene expression profile used in this work is publicly available on NCBI Gene Expression Omnibus (GSE53890): https://www.ncbi.nlm.nih.gov/geo/query/acc.cgi?acc=GSE53890, accessed on 10 September 2018. The RNAseq data used in this work were developed through the Religious Orders Study and Memory and Aging Project (ROSMAP) study [14] and are available at (https://www.synapse.org/#!Synapse:syn3505720, accessed on 10 September 2018) via the AD Knowledge Portal (https://adknowledgeportal.synapse.org, accessed on 10 September 2018). The AD Knowledge Portal is a platform for accessing data, analyses, and tools generated by the Accelerating Medicines Partnership (AMP-AD) Target Discovery Program and other National Institute on Aging (NIA)-supported programs to enable open-science practices and accelerate translational learning. Data is available for general research use according to the following requirements for data access and data attribution (https://adknowledgeportal.synapse.org/#/DataAccess/Instructions, accessed on 10 September 2018).

## Supplementary Materials

The following supporting information can be downloaded at: https://www.mdpi.com/article/10.3390/biom16070992/s1. Supplementary File S1: Hypothetical expression changes in an aging-protective gene across age groups. A. A gene shows increased expression from YO to MO, but loses this trend from MO to OO. B. A gene shows decreased expression from YO to MO but loses this trend from MO to OO. C. A gene shows no significant expression changes from YO to MO, but its expression increases or decreases from MO to OO. Supplementary File S2: DEGs identified between age groups. Supplementary File S3: OO-specific gene co-expression network. Sheet 1: ranked modules in the OO network; Sheet 2: Enriched pathways for the modules in the OO network; Sheet 3: module conservation analysis between the OO and the YO networks. Supplementary File S4: DGCA between age groups. Supplementary File S5: ESEA between age groups. Supplementary File S6: DTGs identified between age groups. Supplementary File S7: AD resilience candidate genes.
