# Supplementary figures and images for: Molecular Networks and Key Regulators Underlying Resilience of the Human Brain to Aging and Dementia
Source: Biomolecules. 2026 Jul 6;16(7):992. doi: 10.3390/biom16070992 (PMC13406847; doi:10.3390/biom16070992)

A

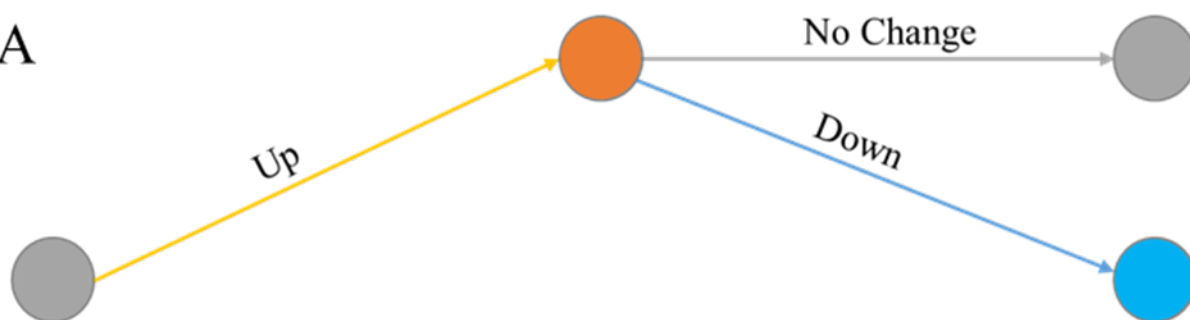

B

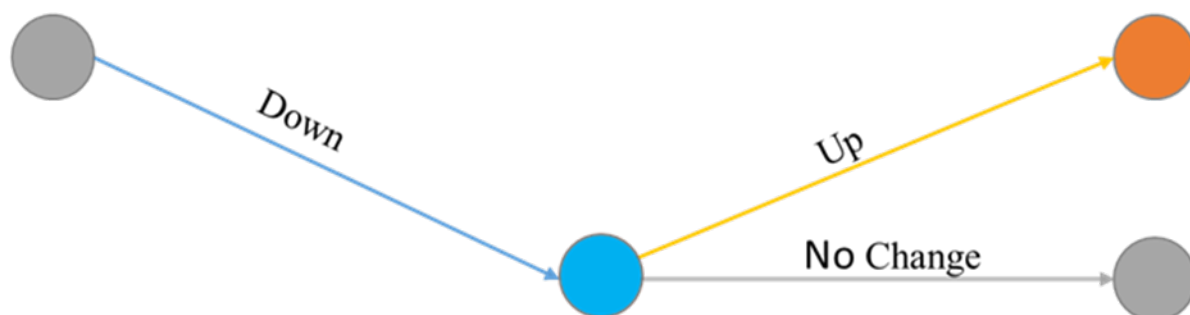

C

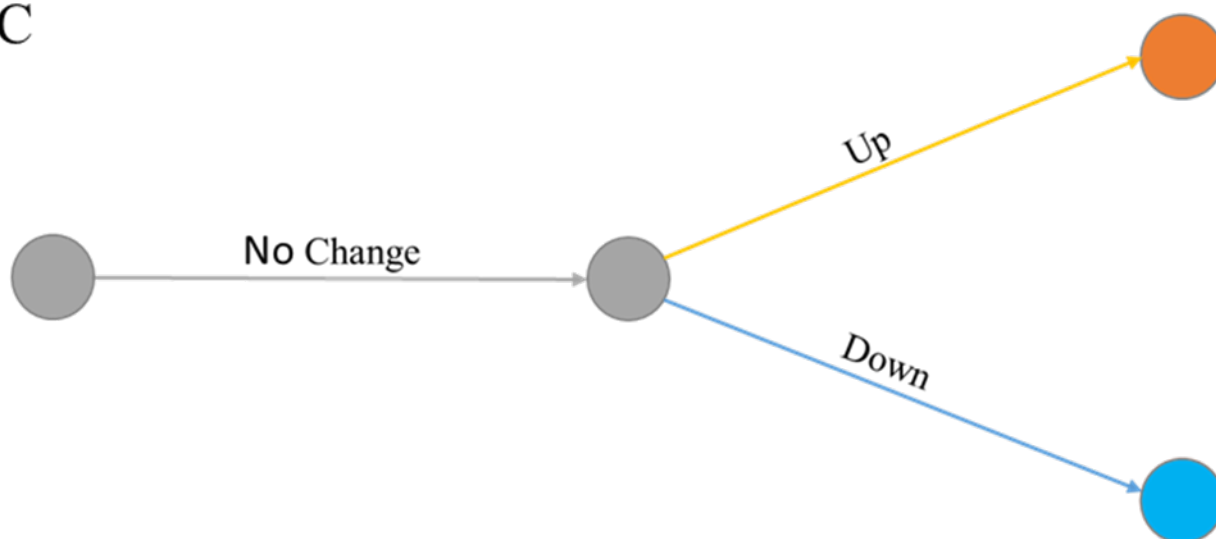

YO

MO

OO

Supplement: Supplementary file 1 [file biomolecules-16-00992-s001.zip › Supplementary File S1.pdf]
